# Trends and Prevalence of Hospital Admissions Related to Deliberate Self-Poisoning and Used Substances in Romanian Adolescents between 2016 and 2022

**DOI:** 10.3390/children10050790

**Published:** 2023-04-27

**Authors:** Elena Predescu, Iulia Calugar, Cristian Bibu-Monus, Roxana Sipos

**Affiliations:** 1 Department of Neuroscience, Psychiatry and Pediatric Psychiatry, “IuliuHatieganu” University of Medicine and Pharmacy, 400489 Cluj-Napoca, Romania; predescu.elena@umfcluj.ro; 2 Clinic of Pediatric Psychiatry and Addiction, Clinical Emergency Hospital for Children, 400489 Cluj-Napoca, Romania; 3 1st Surgical Clinic, Emergency Clinical County Hospital Cluj, 400006 Cluj-Napoca, Romania

**Keywords:** self-poisoning, adolescents, COVID-19, self-harm

## Abstract

Rates of self-poisoning are increasing substantially all around the world, with self-poisoning being the most common form of self-harm leading to hospitalization in children and adolescents. This study aims to investigate the trends in the number of deliberate self-poisoning admissions in Romanian adolescents during the period of 2016–2022, as well as the most frequently used substances and the impact of the COVID-19 pandemic on hospital admissions due to self-poisoning and substance use in relation to these episodes. The sample included 178 patients admitted to the Clinic of Pediatric Psychiatry in Cluj-Napoca from 2016 to 2022 due to an episode of self-poisoning. Data were collected on patients’ sociodemographic characteristics, psychosocial characteristics, and medical histories. We report a slight overall increase in the self-poisoning admission rate during the studied period. There was a decrease during the initial period of the pandemic, with significantly increasing rates in the second year of the pandemic. The mean prevalence rate of hospital admissions due to self-poisoning episodes during the study period was 3.14% (95% CI 2.72, 3.63). Adolescent girls were identified as the most vulnerable group, with the female-to-male ratio increasing dramatically. In terms of substance use, benzodiazepines; over-the-counter analgesics, including paracetamol; and antidepressants were the most frequently used substances. We emphasize the importance of careful consideration in prescribing psychotropic drugs, as well as the need for regulation of over-the-counter drug dispensation.

## 1. Introduction

Deliberate self-poisoning refers to an intentional act of inflicting damage to oneself by ingesting harmful drugs or chemical compounds. This phenomenon has shown an alarming increase over the last 15 years, with self-poisoning being among the most common reasons for emergency hospitalization in adolescents. This also implies potential high risk rates of repeated attempts and readmissions, as well as risk rates of high premature death in the future [1,2]. Increases in self-poisoning rates have been registered globally, with an 96% increase in the number adolescent self-poisonings from 2006 to 2016 in Australia [1], while a 300% increase in the number of self-poisoning calls concerning 10–15-year-olds was registered in the United States from 2000 to 2018 [3]. Moreover, a larger UK population-based cohort study indicated substantial increases in self-poisoning rates for episodes due to paracetamol, non-steroidal anti-inflammatory drugs (NSAIDs), antidepressants, and opioids [4]. The types of substances used for self-poisoning are very important and relevant to developing a better understanding of this difficult medical situation, which can endanger the patient’s life. First, substance toxicity is an important issue, and the most appropriate interventions must be chosen. Secondly, preventive measures can be implemented considering the factors that influence the substance choice. Studies analyzing the substances ingested by adolescents with the intent of self-harm have reported similar results, with the most common substances being paracetamol and NSAIDs, alcohol, antidepressants, opioids, and other psychotropic drugs [2,4,5].

It appears that there are various risk factors linked to self-harm acts, including mental disorders, poor health, adverse childhood experiences [6], bullying [7], low socioeconomic status [5], and the female gender [3,6]. Therefore, it is important to have a complete picture of this high-risk youth category, including the factors and interventions which ensure the best functionality after hospital discharge, to prevent future occurrences of suicidal or self-harm behaviors.

The COVID-19 outbreak has tremendously impacted the population all over the world, raising notable healthcare challenges. Although young people have low susceptibility and a lower risk of developing a severe form of the disease [8,9], it is known that children and adolescents represent a vulnerable age group in regards to mental health [10]. The impact of the COVID-19 pandemic on children and adolescents’ mental health is multifaceted; the available data indicate anxiety, depression, irritability, inattention, and sleep disturbances as the most common issues during this period [11,12,13,14]. Multiple pandemic-related risk factors were identified, such as social isolation and trouble adjusting to online learning, excessive media exposure [14,15], and increased family conflicts and violence [15,16]. Concerns were raised regarding the potential impact of the pandemic on self-harm behavior, with studies showing varying prevalence rates for deliberate self-harm in children and adolescents during this time. The existing data indicate a decrease in the incidence of suicidal behavior during the early period of the COVID-19 pandemic due to numerous factors, which include a reduction in the number of admissions during lockdown [17,18,19,20]. However, later phases of the COVID-19 pandemic, as well as the period following the removal of restrictions, have been associated with increased rates of self-harm and suicide attempts [21].

Although these issues are common, there are limited data on recent trends regarding deliberate self-poisoning and substances used by Romanian adolescents. This study aimed to (1) evaluate trends of deliberate self-poisoning within the aforementioned population during the period of 2016–2022; (2) identify the risk factors related to self-poisoning in adolescents; (3) identify the trend of hospitalizations and use of substances in self-poisoning; and (4) evaluate the impact of the COVID-19 pandemic on admissions due to self-poisoning.

## 2. Materials and Methods

### 2.1. Participants Selection

We conducted a retrospective cohort study on 178 participants aged 11–18 who had been admitted to the Clinic of Pediatric Psychiatry from Cluj-Napoca, Romania, due to an episode of self-poisoning from January 2016 to December 2022. The clinic receives all cases presenting to the emergency service in Cluj-Napoca, as well as the surrounding region.

### 2.2. Data Collection

This study used routinely collected data from the patients’ medical records. Data consisted of researcher-generated variables such as age, gender, living conditions, family conflicts, school difficulties, family history of psychiatric disorders and suicide attempts, previously documented psychiatric treatment, previous suicide attempts, types of drug(s) ingested, days of hospitalization, and the self-poisoning diagnosis based on the 10th revision of the International Statistical Classification of Diseases and Related Health Problems (ICD 10) criteria. The substances used were recorded in five categories—paracetamol, analgesics (including non-opioid analgesics other than paracetamol and nonsteroid anti-inflammatory drugs), psychotropic drugs (antidepressants, antipsychotics, anticonvulsants, benzodiazepines, opioids, and attention deficit/hyperactivity disorder—ADHD-specific medication), ingestion of multiple substances, and other toxic substances (detergents and cleaning products, pesticides and other volatile substances, antibiotics, antispastics, supplements, and other prescription drugs). The study was conducted in accordance with the Declaration of Helsinki. We obtained the approval of the Emergency Clinical Hospital for Children Cluj-Napoca Clinical Trials Quality Assurance Commission (80/14 December 2020).

### 2.3. Statistical Analysis 

The Statistical Package for Social Sciences (SPSS), v. 17, and Microsoft Excel 2019, V. 2302, were used for data analysis. Univariate statistical analysis (mean, standard deviation, and frequencies) was used to describe and assess the selected population. To establish associations among the categorical variables, Spearman correlation analysis was used. For this analysis, we created binary variables to represent the number of days of admission that represented a period shorter than 2 weeks, as well as those that represented periods exceeding 2 weeks. Student’s t-test was used to compare the mean age within the gender groups, as well as the number of self-poisoning presentations per month during the first year of the COVID-19 pandemic relative to the corresponding pre-lockdown period. Similar measurements were applied for the period following lockdown compared to the pre-lockdown period. We compared the differences in mean presentations by month for the seven-year study period using a one-way ANOVA test, applying the Bonferroni post hoc test for within-group analysis. One-way ANOVA was also used to assess the differences in average admission days, as well as age, for each of the X-coded diagnoses. Thus, the number of days of hospitalization and the patient’s age were the dependent variables used for the analysis, while the independent variable was represented by the self-poisoning diagnosis code. Polynomial regression was used to test the presentation trends during the study period. The level of significance was set at 5%, and a confidence interval (CI) of 95% was used for all tests.

## 3. Results

### 3.1. Sample

During the study period, 178 participants were admitted to the Clinic of Pediatric Psychiatry due to self-poisoning. The sample included 147 (82.6%) females and 31 (17.4%) males, with a mean age of 14.94 years old for girls and 15.61 years old for boys. There were no differences in age means within the gender groups (t_176_ = −2.269, *p* = 0.24). The female-to-male ratio was 4.74:1. More than half (55.05%) of the participants were living with their parents, while 39.88% had separated or divorced parents, and 5.05% were living in foster care. A significant number of adolescents (N = 85, 47.75%) reported family conflicts and school difficulties (N = 127, 71.34%). In terms of medical history, 19.66% had a family history of a psychiatric disorder, with 3.93% presenting with a family history of suicide. Following psychiatric assessment at the time of admission, the majority of patients included in the study were diagnosed with depression or anxiety (75.28%), while 21.34% were diagnosed with externalizing disorders (attention deficit/hyperactivity disorder- ADHD/oppositional defiant disorder-ODD/conduct disorder-CD). While admitted, 53.37% of the participants received psychiatric treatment. Moreover, 47.75% of the adolescents presenting with self-poisoning had received previous psychiatric treatment (see Table 1).

### 3.2. Characteristics of Admissions Due to Self-Poisoning

From 1 January 2016 to 31 December 2022, there were a total of 5673 hospital admissions to the Clinic of Pediatric Psychiatry in Cluj-Napoca, of which 178 (3.13%) were due to self-poisoning. These included 110 (61.79%) first-time admissions (see Table 2). 

There was a significant correlation between the history of psychiatric treatment and the first admission due to self-poisoning (r = −0.637, *p* = 0.000); the adolescents with previous psychiatric treatment were less likely to have been admitted for the first due to self-poisoning (OR = 0.39, 95% CI 0.17, 0.93). Moreover, 91.39% of those with no previous psychiatric treatment had been admitted for the first time due to self-poisoning, while 70.5% of those who had received previous psychiatric treatment had a history of multiple admissions to the psychiatry unit due to self-poisoning. Previous psychiatric treatment was also significantly correlated with a history of suicide attempts (r = 0.435, *p* = 0.000), with adolescents who had received psychiatric treatment at some point in time being 8 times more likely to have a history of previous suicide attempts (OR = 8.498, 95% CI 3.88, 18.57). Thus, a significant proportion (81.13%) of adolescents with a history of previous suicide attempts also had a history of psychiatric treatment. There was also a significant correlation between a history of suicide attempts by any method and a first-time admission due to self-poisoning. Adolescents with a history of suicide attempts by any method were significantly less likely to have bee, admitted for the first time due to self-poisoning (OR = 0.125, 95% CI 0.06, 0.25). In addition, a first admission due to self-poisoning was correlated with the duration of hospitalization (r = 0.215, *p* = 0.04); adolescents who had been admitted for the first time due to self-poisoning were 4 times more likely to be admitted for a period shorter than 2 weeks than adolescents with previous presentations due to self-poisoning (OR = 4.097, 95% CI 1.47, 11.37). We found no significant correlation between psychiatric diagnosis and the previously mentioned variables, namely, history of psychiatric treatments, history of suicide attempts by any method, first admission due to self-poisoning, and duration of hospitalization. However, positive correlations were reported between the categorical variables represented by a history of psychiatric treatment and a history of previous suicide attempts (r(176) = 0.435, *p* = 0.00), as well as between the variables referring to a first admission due to self-poisoning and a duration of hospitalization shorter or longer than 2 weeks (r(176) = 0.215, *p* = 0.004). Statistically significant negative correlations were obtained between a first admission due to self-poisoning and both a history of psychiatric treatment (r(176) = −0.637, *p* = 0.00) and a history of previous suicide attempts (r(176) = −0.449, *p* = 0.00). Another negative correlation was observed between the variables referring to a duration of hospitalization shorter/longer than 2 weeks and a history of psychiatric treatment (r(176) = −0.179 *, *p* = 0.17) (see Table 3).

The drugs used were categorized into five groups—paracetamol, analgesics excluding paracetamol, psychotropic drugs, multiple substances, and other substances. The latter group refers to detergents and cleaning products, pesticides, and other volatile substances, including antibiotics, antispastics, supplements, and other prescription drugs (bronchodilators, antihistamines, antihypertensives, antidiarrheals, oral antidiabetics, and levothyroxine). Most self-poisoning episodes are due to the ingestion of multiple drugs (34.83%). However, psychotropic drugs (30.89%) are the most frequently used in self-poisoning episodes in adolescents. Within this group, the most commonly used psychotropic drugs were benzodiazepines (40%), followed by selective serotonin reuptake inhibitors (21.81%) and anticonvulsants (16.36%). Following benzodiazepines, accounting for 12.35% of the total admissions due to self-poisoning was paracetamol, the second-most used drug, which amounted to 10.67% of the total hospital admissions. The ingestion of analgesics was responsible for 5.61% of the total admission, while the “other substances” group accounted for a significant proportion (17.97%) of hospital admissions due to its heterogeneity.

### 3.3. Trends in Self-Poisoning Episodes during the Period of 2016–2022

**Figure 1:**
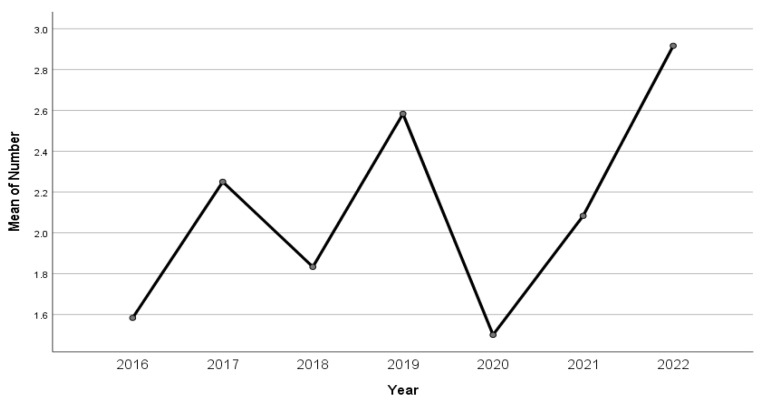
Mean number of presentations by month over the 2016–2022 period.

An overall increase in the number of hospital admissions due to psychiatric illness could be seen starting in 2016, with an overall increase of 20.42% in the number of cases from 2016 to 2019. The number of admissions, however, dropped by 51% in 2020, increasing by 47% in 2021 and with an additional 5.11% in 2022. The number of hospital admissions due to self-poisoning also varied over the 2016–2022 period. We performed a polynomial regression, which indicated that the variability in the number of presentations by month could not be accounted for by either the linear model (r^2^ = 0.000, F(1.82) = 0.00, *p* = 0.99) or the quadratic (r^2^ = 0.019, F(2.81) = 0.77, *p* = 0.46) or cubic models (r^2^ = 0.50, F(3.80) = 1.41, *p* = 0.24). The lowest prevalence in hospital admissions due to self-poisoning was reported in 2016, at a rate of 2.28% (95% CI 1.47, 3.55) of the total yearly hospital admissions, with a mean of 1.58 admissions per month. In comparison, the highest prevalence of hospital admissions due to self-poisoning was reported in 2022, with self-poisoning episodes accounting for a rate of 4.73% (95% CI 3.44, 6.79) and a mean of 2.91 presentations per month. However, there were no statistically significant differences in mean presentations by month between groups when accounting for the seven-year period which was investigated (*p* = 0.353). Moreover, no significant differences were observed within these groups. The reported mean prevalence rate of hospital admissions due to self-poisoning episodes during the study period was 3.14% (95% CI 2.72, 3.63) (see Figure 1).

**Figure 2:**
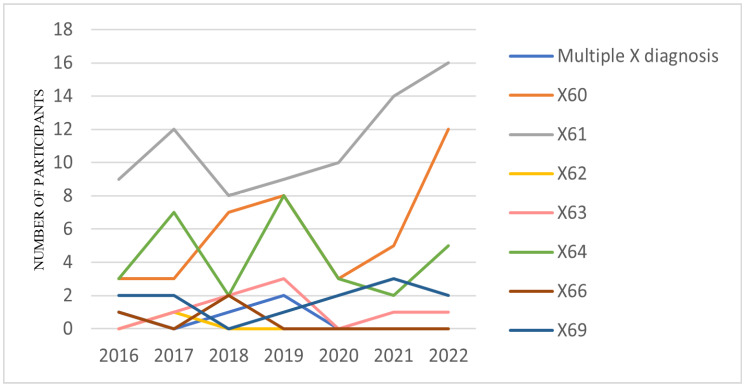
Hospital admissions for self-poisoning from 2016 to 2022, according to ICD 10 codes X60–X69.

Each self-poisoning episode was diagnosed with a X60 (intentional self-poisoning by exposure to nonopioid analgesics, antipyretics, and antirheumatics), X61 (intentional self-poisoning by exposure to antiepileptic, sedative–hypnotic, antiparkinsonism, and psychotropic drugs, not elsewhere classified), X62 (intentional self-poisoning by exposure to narcotics and psychodysleptics (hallucinogens), not elsewhere classified), X63 (intentional self-poisoning by exposure to other drugs acting on the autonomic nervous system), X64 (intentional self-poisoning by exposure to other unspecified drugs, medicaments, and biological substances), X65 (intentional self-poisoning by exposure to alcohol), X66 (intentional self-poisoning by exposure to organic solvents, halogenated hydrocarbons, and their vapors), X67 (intentional self-poisoning by exposure to other gases and vapors), X68 (intentional self-poisoning by exposure to pesticides), or X69 (intentional self-poisoning by exposure to other, unspecified chemicals and noxious substances) code, according to the ICD-10 criteria [22]. The most frequent diagnosis codes were X61 (N = 78, 43.82%), followed by X60 (N = 41, 23.03%) and X64 (N = 30, 16.85%) (see Figure 2). 

An one-way analysis of variance found no statistically significant differences in mean days of hospitalization between the diagnostic groups (F(8,169) = 0.51, *p* = 0.819). Similarly, we found no significant differences in age means by diagnosis group (F(8,169) = 1.14, *p* = 0.338).

**Figure 3:**
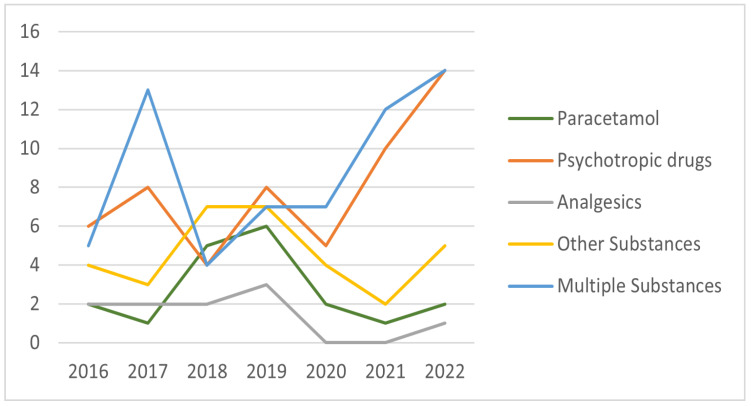
The most common substances used for self-poisoning from 2016 to 2022.

Although the ingestion of paracetamol, analgesics, and drugs included in the other substance categories presented a steady increase in occurrence from 2017–2019, the number of admissions due to the ingestion of these substances decreased during the 2020–2021 pandemic period. Mirroring this situation, the ingestion of psychotropic and multiple substances presented a descending trend during the initial study period of 2018–2019, while these instances steadily increased in occurrence after the onset of the COVID-19 pandemic in 2020 (see Figure 3).

### 3.4. The Impact of COVID-19 Pandemic

**Figure 4:**
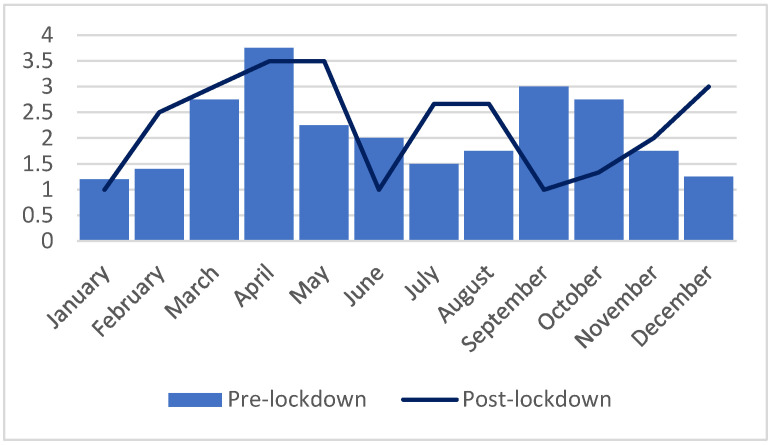
Mean number of admissions per month during the pre-lockdown period (January 2016–February 2020) compared to the post-lockdown period (June 2020–December 2022).

In March 2020, Romania declared a state of emergency due to the COVID-19 pandemic, followed by national lockdown which lasted until May 2020. The year 2020 registered the lowest number of hospital admissions (N = 489) during the studied period, with 3.86% (N = 18) being due to self-poisoning episodes. In 2020, the female-to-male ratio decreased from 3.4:1 in the previous year to 2.6:1. Following the period after lockdown, an increase in the number of hospital admissions was registered in 2021 (N = 723), with 3.45% (N = 25) of admissions being due to self-poisoning. The female-to-male ratio increased to 11.5:1 (see Figure 4).

We compared the mean number of hospital admissions per month during the first year of the COVID-19 pandemic (March 2020–February 2021) relative to the year prior to the pandemic (March 2019–February 2020). The mean number of admissions during the first year of the pandemic was significantly lower than the year prior to its onset (t_22_ = −2.20, *p* = 0.03; 95% CI −3.1, −0.22; Cohen’s d = 3.12). However, comparing the mean number of hospital admission per month during the period preceding the lockdown (January 2016–February 2020) with the mean number of admissions per month in the period following the lockdown (April 2020 to December 2022), no statistically significant difference was found (t_22_ = −0.4, *p* = 0.67).

## 4. Discussion

Deliberate self-poisoning is the most common cause of hospital admissions for self-harm in children and adolescents [23], and has registered a significant increase in recent years [1]. Within the selected sample, self-poisoning episodes were largely associated with gender, with adolescent girls accounting for 82.6% of the participants in the study. Moreover, 32.6% of the female subjects had a history of previous suicide attempts. It has been widely pointed out that female gender is a risk factor for self-harm behavior, as well as for repeated self-harm episodes [24]. A plausible explanation is the fact that adolescent girls are at higher risk to suffer from depression, with rising prevalence rates being evident over the past decade [25], which significantly increases the risk of self-harm and suicide attempts [26]. One-third of the adolescent girls included in the study had internalizing disorders (depression/anxiety), while almost a quarter were diagnosed with externalizing disorders (ADHD/CD/ODD). Regarding the externalizing disorders, impulsivity and emotional dysregulation play an important role in increasing the risk of self-harm [27,28,29]. Similar findings were reported for the adolescent boys included in the study, although their numbers were significantly lower. Given the frequent association between self-poisoning and the psychiatric pathology, it is recommended to carry out a complete assessment in these cases. Furthermore, psychiatric morbidity and previous self-harm attempts have been shown to be involved in self-harm repetition [24]. Some authors have considered that longitudinal studies on the progress of these disorders may be required [18]. Our study supports the findings regarding the association between self-poisoning and psychiatric disorders, but most often, deliberate self-poisoning in adolescence represents a complex condition in which individual risk factors are interacting with psychological, sociodemographic, and other social factors [24]. Moreover, a family history of mental health issues is another notable risk factor associated with suicidal behavior in children [30].

In terms of psychosocial risk factors, self-harm by self-poisoning is linked to school activity, with increased occurrence during the school periods [31,32] due to school-related stressors. Parental divorce [33], exposure to interpersonal violence [34,35], family conflicts [36], and low socioeconomic status [35] are also associated with a higher prevalence of self-poisoning. Our results indicate that a significant proportion of the study participants presented with school difficulties and conflictual family relationships, or came from single-parent families.

This study demonstrates the overall increase in hospital admissions due to self-poisoning over the studied period. The data indicate an ascending trend worldwide [37], suggesting that self-harm is associated with a significant disease burden in adolescents all over the world. The onset of the COVID-19 pandemic was associated with a reduction in the number of self-poisoning hospital admissions. Our findings were consistent with studies from all over the world. A UK comparative trend analysis on self-harm hospital presentations during the early period of the COVID-19 pandemic indicated a marked reduction in numbers following lockdown introduction, especially on account of self-harm by self-poisoning [38]. Similarly, a decrease in presentations due to suicidal behavior in the incipient period of the COVID-19 pandemic was reported in studies from the US, France, and Spain [39,40,41]. The precise reasons for this phenomenon are unclear; however, reduced access to health services, fear of exposure, and social distancing, alleviating stressors associated with suicidal behavior, might be contributing factors. However, further research into this subject is needed to gain a better understanding of the relationship between the pandemic and the reduction in self-poisoning episodes. Nonetheless, the COVID-19 pandemic has had a great impact on adolescents’ mental health due to social isolation and difficulties adapting to online learning, exposure to potentially harmful home environments, and disruption of daily activity [15]. As the pandemic continued, the number of hospital admissions due to suicidal behavior increased substantially. Our results support this change in the trend. A German study conducted on pediatric ICU (Intensive Care Unit) admissions due to adolescents’ suicide attempts during the pandemic showed a decreased number of hospital admissions during the first wave of the COVID-19 pandemic, with a dramatic increase during the second lockdown in 2021; drug ingestion was responsible for almost half of the admissions [21]. We also found that adolescent girls were at higher risk for self-harm and suicidal behavior after the initial wave of COVID-19, with the female-to-male ratio increasing substantially (11.5:1). Similar results were reported by a Spanish study conducted on a population of adolescents admitted to the Psychiatric Emergency Department of Clinical Hospital of Barcelona. Self-harm admissions registered a 177% rise in numbers, with the female gender being a significant risk factor for these admissions [42]. The comparison of monthly admissions due to self-poisoning did not differ significantly between the pre-lockdown period and the post-lockdown period within our sample. However, the long-term effects of the pandemic on mental health are still relatively unknown.

Regarding the substances used for self-poisoning, we identified psychotropic drugs as the most commonly used class. This finding is not unexpected, as prescriptions for psychotropic agents have increased worldwide [43]. An Australian cohort study on trends in self-poisoning and psychotropic drug use in patients aged 5–19 years has demonstrated significant increases in the prescription of psychotropic drugs, especially antidepressants and antipsychotics [1]. Benzodiazepines were the most frequently used within our study, amounting to 12.35% of the self-poisoning admissions. Most patients had already been prescribed psychiatric treatment, this situation ensuring the availability of and easy access to this class of medication. A recent study on child and adolescent benzodiazepine exposure and overdose was conducted in the US, using data from 2000 to 2015. Over the studied period, exposure to benzodiazepines increased by 54%, as did the severity of the outcomes and the prevalence of multiple drug ingestion. We report similar results, as multiple substance ingestion accounted for the largest portion of hospital admissions due to self-poisoning [44]. Furthermore, the use of psychotropic medication raised concerns regarding a potentially increased risk of suicidal ideation and self-harm [45].

Our study also found paracetamol to be frequently used in self-poisoning presentations, being responsible for 10.67% of the hospital admissions. Analgesics, excluding paracetamol, were found to be another well-represented substance class. There is a large body of literature indicating that over-the-counter analgesics are the most common substances involved in self-poisoning in young people [1,4,46,47,48]. During the studied period, the ingestion of analgesics, including paracetamol, showed an increase from 2017 to 2019, but slowly decreased over the pandemic period. Possible contributing factors could involve the restrictions during the pandemic, which reduced access to over-the-counter medication and increased parental supervision. Mirroring this situation, use of psychotropic medication significantly increased during the pandemic. This is probably due to the fact that most people who deliberately self-poison use medication that has previously been prescribed to them [49].

## 5. Conclusions

There has been an overall increase in hospital admissions due to self-poisoning in children and adolescents from 2016 to 2022, with a mean prevalence of 3.14% (95% CI 2.72, 3.63). Although there was a decrease in numbers in the incipient period of the COVID-19 pandemic, they rose rapidly after the first wave of COVID-19. Even though presentations due to self-poisoning clearly rose during this period, we found no statistically significant differences between pre-lockdown monthly admissions and post-lockdown ones. However, there is a need for further research investigating the relationship between self-harm behavior and the long-term consequences of COVID-19 on mental health, as they are still relatively unknown. Prevention efforts may include promoting healthy coping strategies, reducing access to harmful substances, and increasing access to mental health resources. It is essential to create a safe and supportive environment for adolescents to seek help, and to address the underlying issues that may be contributing to self-poisoning. The most frequently used substances during the studied period were benzodiazepines, paracetamol, and antidepressants. Thus, physicians should carefully consider the prescription of psychotropic drugs, especially to patients considered to be at risk for self-poisoning. Moreover, legislation and regulations should be considered for over-the-counter drug commercialization.

## Figures and Tables

**Table 1:** Demographics and characteristics, stratified by gender.

Characteristics		F (N = 147)	M (N = 31)	Total (N = 178)
Age		14.94 (SD 1.52)	15.61 (SD 1.38)	15.06 (SD 1.52)
Living with both parents		78	20	98 (55.05%)
Divorced parents		60	11	71 (39.88%)
Foster home		9	0	9 (5.05%)
Family psychiatric history		33	2	35 (19.66%)
Family suicide history		7	0	7 (3.93%)
Psychiatric diagnosis Depression/anxiety		112	22	134 (75.28%)
Psychiatric diagnosis ADHD/ODD/CD		29	9	38 (21.34%)
Psychiatric treatment		77	18	95 (53.37%)
History of psychiatric treatment		68	17	85 (47.75%)
History of previous suicide attempts		48	5	53 (29.77%)
Substances used for self-poisoning	Paracetamol	16	3	19 (10.67%)
	Analgesics, excluding paracetamol	8	2	10 (5.61%)
	Psychotropic drugs	45	10	55 (30.89%)
	Multiple substances	50	12	62 (34.83%)
	Other substances	28	4	17.97%)
School difficulties		105	22	127 (71.34%)
Family conflicts		70	15	85 (47.75%)

**Table 2:** Admissions due to self-poisoning by year and percentage of total admissions of children and adolescents to the psychiatric unit during the period of 2016–2022.

Year of Admission	2016	2017	2018	2019	2020	2021	2022	Total
Total admissions	830	914	959	998	489	723	760	5673
Total admissions due to self-poisoning, N (%)	19 (2.28%)	27 (2.9%)	22 (2.29%)	31 (3.1%)	18 (3.68%)	25 (3.45%)	36 (4.73%)	178 (3.14%)
First-time admission N (%) due to self-poisoning	13 (68.42%)	23 (85.18%)	18 (81.81%)	11 (35.48%)	10 (55.55%)	12 (48%)	23 (63.88%)	110 (61.79%)

**Table 3:** Spearman’s correlation coefficients concerning the adolescents’ psychiatric history and characteristics at admission.

	1	2	3	4	5
1. Psychiatric diagnosis	1				
2. History of psychiatric treatment	0.012	1			
3. History of previous suicide attempts	0.001	0.435 **	1		
4. First admission due to self-poisoning	0.171 *	−0.637 **	−0.449 **	1	
5. Duration of hospitalization (shorter/longer than 2 weeks)	−0.25	−0.179 *	−0.093	0.215 **	1

*. Correlation is significant at the 0.05 level (2-tailed). **. Correlation is significant at the 0.01 level (2-tailed).

## Data Availability

The data presented in this study are available upon request from the corresponding author. The data are not publicly available due to privacy and ethical considerations.
